# Complete chloroplast genome sequence of *Chimonanthus praecox* link (Calycanthaceae): an endemic plant species in China

**DOI:** 10.1080/23802359.2020.1823277

**Published:** 2020-10-05

**Authors:** Guang-Can Zhou, Jie-Yu Wang, Wen Li, Ming Zhang, Guo-Qing Meng, Heng-Yi Wang, Xiao Chen, Yu-Han Wu, Ping Wu, Yi-Lei Wang

**Affiliations:** a College of Agricultural and Biological Engineering (College of Tree Peony), Heze University, Heze, China; b Key Laboratory of Plant Resources Conservation and Sustainable Utilization, South China Botanical Garden, Chinese Academy of Sciences, Guangzhou, China; c National Facility for Protein Science in Shanghai, Shanghai Advanced Research Institute, Chinese Academy of Sciences, Shanghai, China

**Keywords:** *Chimonanthus praecox*, complete chloroplast genome, Illumina sequencing, Calycanthaceae, phylogeny

## Abstract

*Chimonanthus praecox*, a deciduous shrub tree, is endemic to China and widely cultivated in the world as a popular garden and ornamental plant. Here, we have reported its complete chloroplast genome with a length of 153,181 bp, containing a large single copy (LSC) region of 86,916 bp, a small single copy (SSC) region of 19,767 bp and two identical inverted repeat regions (IRs) of 23,249 bp. The overall GC contents of the plastome were 39.27%. A total of 114 unique genes were successfully annotated consisting of 80 protein-coding genes, 30 tRNA genes and four rRNA genes. Sixteen genes each possessed one intron and three genes had two introns. The ML phylogenetic analysis supports *Chimonanthus* as sister to *Calycanthus*. This result will be helpful for genetic breeding and population genetics of *C. praecox*, DNA barcoding of *Chimonanthus*, and phylogenetic studies of Calycanthaceae.

*Chimonanthus praecox* (linn.) Link, a deciduous shrub tree, is one of six species of the genus *Chimonanthus* native to China (Zhou et al. 2006). Now this species is widely cultivated in China and other temperate areas of the world as a popular garden and ornamental plant. *Chimonanthus praecox* is also used for medicine purposes, due to its extracts exhibiting multi-bioactivities (Ueyama et al. 1990; Zhang et al. 2009; Wang et al. 2011; Lou et al. 2018; Shu et al. 2019). In this study, we report and characterize the complete chloroplast genome of *C*. *praecox* might provide remarkable information for its molecular phylogeny and genetic breeding.

Fresh young leaves of *C. praecox* were collected from the same individual growing in the Institute of Botany, Jiangsu Province and Chinese Academy of Sciences (32.06˚N, 118.84˚E). A voucher specimen numbered FCM05 was deposited at the Herbarium of Institute of Botany, Jiangsu Province and Chinese Academy of Sciences. Genomic DNA was extracted from approximately 100 g fresh leaves followed the method of Ahmed and Fu (2015). The DNA concentration and quality were assessed using a Qubit fluorometer (Invitrogen, San Diego, CA, USA) and a NanoDrop Spectrophotometer ND-1000 (NanoDrop Technologies, Wilmington, DE, USA), respectively, and then sequenced by Illumina Hiseq 2500 Sequencing platform (Illumina, Hayward, CA). Raw sequence reads have been deposited in the European Nucleotide Archive under BioProject ID PRJEB33250 (Accession no. ERS3550534). The generated raw data were *de novo* assembled using the program CLC Genomics Workbench v6.5 (CLC Bio, Aarhus, Denmark) with default parameters. The chloroplast genome was reconstructed by blasting all contigs against confamilial species *Calycanthus floridus* chloroplast genome (Accession no.: NC_004993) with the CLC BLAST tool. Annotations of the complete chloroplast genome of *C. praecox* were carried out using the online program GeSeq according to default values (Tillich et al. 2017), with *Ca. floridus* (Accession no.: NC_004993) as the reference, and adjusted manually in Geneious v6.0.3 (Biomatters Ltd, Auckland, New Zealand).

The annotated chloroplast genome was deposited at the GenBank database (Accession no. MT859152). The size of chloroplast genome was 153,181bp with the typical plastome structure of land plants, containing a large single copy (LSC) region of 86,916 bp, a small single copy (SSC) region of 19,767 bp and two identical inverted repeat regions (IRs: A and B) of 23,249 bp. However, the chloroplast genome of *C. praecox* was 71 bp smaller than that of Zhou’s submission (Accession no. NC_042744; Direct Deposit in GenBank). Additionally, 154 SNPs and 15 indels were found between the two *C. praecox* chloroplast genome sequences. Among the 154 SNPs, 58 SNPs were located in the exons of *psaB* (11), *psaA* (7), *ycf2* (8), and *ndhB* (32); one SNP was located in the intron of *trnS-CGA*; 91 SNPs were located in the intergenic regions of *ycf2-trnL-CAA* (42), *trnL-CAA-ndhB* (34), *trnA-UGC-rrna23* (14), and *rps15-ycf1* (1); and four SNPs were located in *trnL-CAA.* There were 12 indels in introns, while only three indels in protein-coding genes (*psaB* and *ycf2*).

The overall GC content of the plastome is 39.27%, in which the corresponding values of the LSC, SSC, and IR region are 38.14%, 33.97%, and 43.65%, respectively. A total of 114 unique genes were successfully annotated consisting of 80 protein-coding genes, 30 tRNA genes and four rRNA genes. Among these genes, 16 genes (10 protein-coding and six tRNA genes) possessed one intron, while three genes (*rps12*, *ycf3*, *clpP*) had two introns. The *rps12* gene was trans-spliced, with the 5′ exon located in the LSC region, and the 3′ exons duplicated in the IR region.

We used the complete chloroplast genome sequence of *C. praecox* and other 23 plastomes to construct a Maximum Likelihood tree through IQ-TREE based on the best-fit model estimated by ModelFinder (Nguyen et al. 2015; Kalyaanamoorthy et al. 2017); branch support values were assessed using UFBoot2 tests (Minh et al. 2013). The phylogenetic analysis indicated that *C. praecox* and *C. nitens* formed a clade of *Chimonanthus*, and the clade was subsequently sister to *Calycanthus* (Figure 1), which is consistent with a previous study (Zhou et al. 2006). The new plastome sequence would provide a useful resource for genetic breeding and population genetics of this species, DNA barcoding of *Chimonanthus*, and the phylogenetic studies of Calycanthaceae.

**Figure 1.**
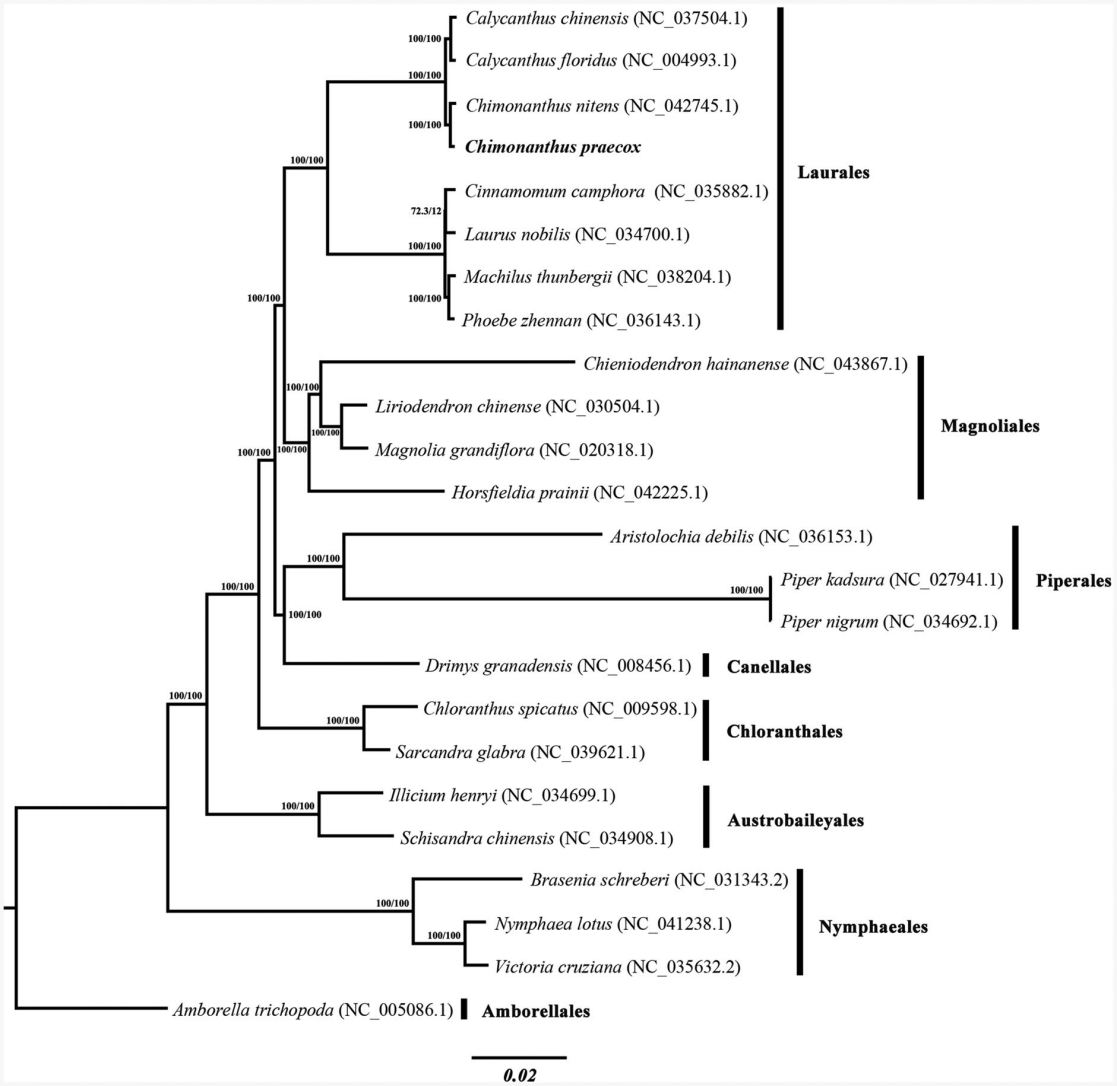
Maximum-likelihood phylogenetic tree based on 24 complete chloroplast genome sequences, with *Amborella trichopoda* as outgroup. The new plastome obtained in this study is shown in bold. The numbers at each node are SH-aLRT support (%)/ultrafast bootstrap support (%) values.

## Data Availability

The data that support the findings of this study are openly available in the European Nucleotide Archive under BioProject ID PRJEB33250 with accession No. ERS3550534 and GenBank of NCBI at https://www.ncbi.nlm.nih.gov/, reference number MT859152.
